# Identification of key genes associated with mesocotyl length through a genome-wide association study in rice

**DOI:** 10.3389/fpls.2025.1546580

**Published:** 2025-05-13

**Authors:** Li Xue, Sen Wang, Qiuyu Zhang, Bing Han, Di Cui, Longzhi Han, Jianxin Deng, Xiaoding Ma

**Affiliations:** ^1^ College of Agriculture, Yangtze University, Hubei, China; ^2^ State Key Laboratory of Crop Gene Resources and Breeding, Institute of Crop Sciences, Chinese Academy of Agricultural Sciences, Beijing, China; ^3^ National Key Laboratory, Sichuan Agricultural University, Sichuan, China; ^4^ Chongqing Engineering Research Center of Specialty Crop Resources, Chongqing Normal University, Chongqing, China

**Keywords:** rice, mesocotyl length, genome-wide association analysis, haplotype, candidate gene

## Abstract

Mesocotyl length is a key trait affecting seedling emergence and establishment in dry direct-seeded rice, with longer mesocotyls promoting rapid and uniform emergence, thereby forming larger effective populations. Therefore, mining genes associated with mesocotyl length will facilitate the development of rice varieties suitable for dry direct seeding. In this study, 300 rice germplasm resources with a wide range of sources were selected as experimental materials. Phenotypic traits such as mesocotyl length and seedling emergence rate were systematically determined in each variety by setting different mulch depth treatments. Genome-wide association analysis (GWAS) was used to locate QTL controlling mesocotyl length and predict candidate genes. The results showed that mesocotyl length increased significantly with greater soil cover depth, while excessively deep sowing treatments inhibited seedling emergence. The GWAS analysis identified four QTLs associated with mesocotyl length and two QTLs associated with seedling emergence, with phenotypic contributions of 6.96-8.48%. Among them, the mesocotyl length-related QTL *qML3* located at 28.03-28.43 Mb on chromosome 3 was detected at both sowing depths. Gene annotation analysis identified nine candidate genes related to plant hormones and transcription factors for *qML3*. Further investigation revealed three genes (*LOC_Os03g49250*, *LOC_Os03g49400*, and *LOC_Os03g49510*) exhibiting distinct haplotypes with significant differences in mesocotyl length, suggesting they may be causal genes for *qML3*. The results provide new clues to elucidate the molecular mechanism of rice mesocotyl development and lay an important foundation for subsequent gene function verification and molecular breeding. In the future, the functions of these candidate genes will be verified by transgenic and other methods, and molecular markers will be developed for genetic improvement of drought-tolerant rice varieties.

## 1 Introduction

Rice (*Oryza sativa* L.) is a major global food crop that is traditionally grown by transplanting seedlings into paddy fields (Zhao et al., 2018). However, conventional transplanted rice production systems face significant challenges because of climate change, urbanization, water scarcity, and labor shortages in many regions (Sagare et al., 2020; Zhang et al., 2023). Dry direct seeding, as an alternative to transplanting, saves time and labor while reducing water and energy consumption (Liu et al., 2019; Ohno et al., 2018; Zhan et al., 2019). Additionally, direct dry seeding eliminates the transplanting step, not only accelerating seedling cultivation but also increasing yield (Ishfaq et al., 2018; Muhammad et al., 2020). Although direct seeding of rice has multiple advantages over traditional planting methods, its popularization still faces several challenges, such as a low seedling emergence rate (ER), poor seedling establishment, weed infestation, and susceptibility to lodging (Mahender et al., 2015; Lee et al., 2017). Therefore, a key challenge at this stage is to identify effective methods to promote rapid and high-quality germination and growth of direct-seeded rice.

The mesocotyl, which forms when rice seeds germinate in the dark, is the organ that connects the germinal sheath to the seed root (Schillinger et al., 1998; Zhang et al., 2012). Light is a key factor influencing mesocotyl length (ML). Typically, light inhibits ML, whereas darkness significantly promotes its elongation. The study revealed that under light-free conditions, the cells of the mesocotyl not only actively divided but also exhibited particularly pronounced elongation and growth. This process is accompanied by increased levels of phytohormones such as gibberellins (GA), auxins (IAA), and cytokinins (CK), which act synergistically to strongly promote mesocotyl elongation (Li et al., 2012, 2013). In addition, temperature also affects the elongation of the ML. The optimal temperature range is 25-30°C, within which the ML increases with the rise in temperature. Beyond the suitable temperature range, as the temperature continues to rise, the growth rate of the rice mesocotyl slows down (Cao et al., 2002). Temperature is another factor that regulates the elongation of the mesodermal axis. The optimal temperature promotes growth, while both high and low temperatures are detrimental to the elongation of the mesodermal axis. Thus, the mesocotyl is not only a central feature of seed germination adapted to dark conditions but also a crucial factor in regulating the initial morphogenesis and growth strategy of rice seedlings. Rice seeds with longer mesocotyls demonstrate superior seedling emergence and uniformity (Turner et al., 1982; Mgonja et al., 1993; Chung, 2010). ML is recognized as a key trait for breeding direct-seeded rice varieties (Zhan et al., 2019). Therefore, an economical and effective method to promote the adoption of direct seeding technology is to investigate the locus ML, analyze the underlying genetic mechanisms, and screen and innovate germplasm with long mesocotyls.

ML can be influenced by external environmental factors but is also determined by intrinsic genetic mechanisms (Gray et al., 1998; Kato and Katsura, 2014; Liu et al., 2023). ML is typically a quantitative inherited trait controlled by multiple genes (Wu et al., 2005; Wang et al., 2021; Zhang et al., 2023). Therefore, mining of ML-related genes and molecular breeding could facilitate the development of direct-seeded rice varieties. With the rapid development of molecular marker technology and genome sequencing technology, many ML-related QTLs have been identified in various natural populations through biparental QTL analysis and genome-wide association studies (Lee et al., 2017; Liu et al., 2019; Zhan et al., 2019; Liu et al., 2023; Wang et al., 2023);. Eleven QTLs were identified using a population of recombinant inbred lines (RILs) from an interspecific cross between *O. sativa* and *O. rufipogon* (Cai and Morishima, 2002). In a population of backcrossed inbred lines (BILs) from a cross between Kasalath and Nipponbare, five QTLs for ML were detected (Lee et al., 2011). Three major QTLs, designated *qMel-1*, *qMel-3*, and *qMel-6*, located on chromosomes 1, 3, and 6, respectively, were identified through the analysis of 98 self-crossed lines from the Kasalath and Nipponbare crosses (Lee et al., 2017). In addition, 9, 17, 16, and 11 QTLs for ML were identified using GWAS (Wu et al., 2015; Lu et al., 2016; Liu et al., 2019; Kwon, 2021). To date, several ML-related genes have been cloned. Xiong et al. (2017) reported that ethylene (ETH) regulates ML by repressing *GY1* gene expression and controlling the biosynthesis of jasmonic acid (JA). Sun et al. (2018) reported that the *OsGSK2* gene regulates ML by coordinating the signaling of strigolactone (SL) and brassinolide (BR). Zheng et al. (2020) reported that the karrikin signaling pathway and the BR signaling pathway may jointly regulate mesoembryo axis elongation.

ML as a crucial indicator of rice growth and development, demonstrates significant pleiotropic effects through its regulatory genes in crop breeding. Choi (2003) reported that *OsEXP4* overexpression produced a biphasic phenotype with 12% of plants showing increased height and 88% displaying reduced stature compared to controls; the overexpressing lines exhibited 31% and 97% increases in coleoptile and ML, respectively, while antisense plants showed 28% and 43% reductions. Ma et al. (2013) found that ethylene treatment significantly promoted coleoptile and mesocotyl elongation in etiolated *MHZ7* overexpressing plants while increasing grain length in transgenic plants, whereas *mhz7* mutants exhibited reduced thousand-grain weight. Lv et al. (2021) demonstrated that *OsPAO5* knockout across different genetic backgrounds significantly enhanced mesocotyl elongation, improved emergence rate and speed under deep-sowing conditions, and concurrently increased grain length, grain weight, and grains per panicle, ultimately boosting single-plant yield. Through analysis of 165 rice accessions, Meng et al. (2023) further identified the superior gene *OsML1*, which not only markedly improved emergence rate in deep sowing but also conferred comprehensive enhancements in agronomic traits including plant height, panicle length, and grain width in overexpression lines.

Discovering rice resources with long mesocotyls, identifying and cloning the genes that control ML, and analyzing their molecular regulatory mechanisms are key steps in breeding rice varieties with long mesocotyls. In this study, a GWAS was conducted on a rice population consisting of 300 genotypes to assess mesocotyl phenotypes. Four new QTLs were ultimately identified, and three new candidate genes for the ML were initially predicted. This study lays the foundation for the cloning of new ML genes and analyzing the genetic mechanisms of rice ML.

## 2 Materials and methods

### 2.1 Plant materials

In this study, we selected 300 rice varieties, all of which were derived from the National Gene Bank of China. Based on previous research results, we selected samples including local varieties, superior varieties, foundation varieties and their derivatives to ensure a wide distribution and high genetic diversity. (**Supplementary Table S1**) (Cui et al., 2022). These accessions were grown in Hainan Province, China, in 2022. The seeds were harvested in April 2023, air-dried, and stored in the laboratory of the Institute of Crop Sciences, Chinese Academy of Agricultural Sciences, Beijing, China.

### 2.2 Mesocotyl length measurement and emergence rate evaluation

Seeds were placed in an oven at 45°C for one week to break dormancy. For each treatment, 50 full and uniform seeds were independently selected and sterilized with 1.4% sodium hypochlorite solution and then placed in an oven at 35°C for 2 days to promote germination. To assess the effect of different sowing depths on mesocotyl length (ME), three mulching depth treatments of 2, 5 and 7 cm were set up in 84-well plastic frames, with three biological replicates at each depth, and 20 high-quality germinated seeds were sown in each replicate, and then incubated for 7 days in the dark at 28°C. The mesocotyl length of each seedling was measured using a ruler (distance from root base to the node of the embryonic sheath) (Huang et al., 2022). The plates were incubated in a 28°C dark growth chamber for 7 days, after which the mesocotyl length (ML) was measured. For the emergence rate (ER), germinated seeds were sown at depths of 5 cm and 7 cm respectively, and cultivated under conditions of 30°C and 75% relative humidity with 20,000 lux white fluorescent lighting (14 h light/10 h dark photoperiod) for 7 days. All experiments included three biological replicates, with at least 10 seedlings measured per replicate. Phenotypic variation analysis, correlation analysis, and Student’s t-tests were performed using IBM SPSS v26.

### 2.3 Genome-wide association study and candidate genes identification

SNPs with a minor allele frequency (MAF) greater than 5% and deletion under 40% were selected for association analysis, and 711,268 variants were used for GWAS. The Yeo–Johnson transformation was applied to normalize the data for GWAS analysis (Yeo and Johnson, 2000). The GWAS was performed using a mixed linear model (MLM) and the script run_pipeline.pl in the TASSEL software (Bradbury et al., 2007). The significance threshold was set at 1.0 × 10^-7^. Principal component analysis (PCA) was used to characterize and differentiate the samples using Plink software (Purcell et al., 2007). SNP density heatmaps and Manhattan and Q–Q plots were generated using the CMplot package in R software (Yin et al., 2021).

QTLs were screened based on the results of the Manhattan plot, where column peaks exceeding the threshold line were considered as candidate QTLs. According to existing studies, genome-wide linkage disequilibrium (LD) attenuation in cultivated rice usually occurs in the range of 100–200 kb. Therefore, we designated the 200 kb region upstream and downstream of the QTL peak SNP locus as the QTL region. We extracted the genes encoding the target intervals from the Rice Expression Database (http://expression.ic4r.org/) and screened them using gene function annotation information from the China Rice data Center (https://ngdc.cncb.ac.cn/red/index). First, transposon- and reverse transcript-transposon-related genes were excluded, and then candidate genes related to the target QTL were preliminarily screened based on the gene functional annotations in combination with existing research reports on rice mesocotyl and internode elongation genes (Kawahara et al., 2013).

### 2.4 Haplotype analysis

Haplotype analyses were performed on the QTL candidate genes that were repeatedly detected under two different environmental treatments. High-confidence SNPs in the coding regions of the candidate genes within the QTL regions were extracted from the Rice Variation Atlas v2.0 database (http://ricevarmap.ncpgr.cn/) (Zhao et al., 2021), and the haplotypes of the candidate genes within the QTL regions were analyzed. The significance of the differences between the phenotypic values and haplotypes was determined by independent samples t-test (P<0.05), and genes showing significant differences in phenotypic value between the different haplotypes were screened by comparing the phenotypic values of the different haplotypes (≥10 materials).

## 3 Results

### 3.1 Phenotypic variation

**Figure 1:**
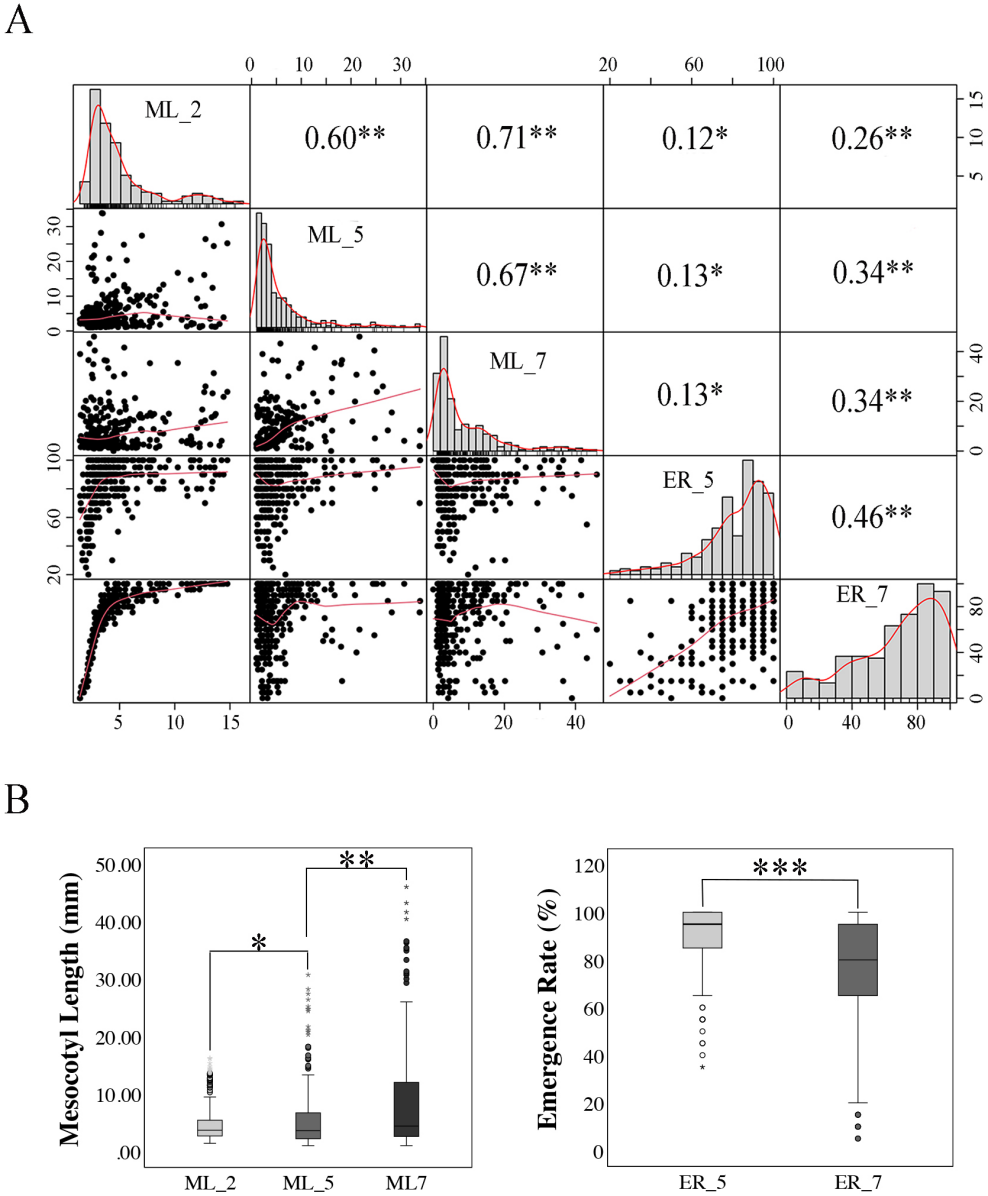
Correlation and differential analysis of mesocotyl length and emergence rate under three deep sowing depths. **(A)** Phenotypic distribution and correlation analysis of mesocotyl length and emergence rate under 2cm, 5 cm and 7 cm deep sowing conditions. Numbers in upper right corner are correlation coefficients (r), * indicates the significance at p ≤ 0.05. **(B)** Phenotypic variations of phenotypic traits: mesocotyl length and emergence rate under 5 cm and 7 cm deep sowing conditions. Significance levels are indicated by asterisks: *P < 0.05, **P < 0.01, ***P < 0.001 (two-tailed *t-test*). ML_2, ML_5 and ML_7: mesocotyl length under deep sowing depths of 2cm, 5 cm and 7 cm; ER_5 and ER_7: emergence rate under deep sowing depths of 5 cm and 7 cm.

Firstly, the ML of 300 rice materials was evaluated under different overburden conditions (2, 5, 7cm). The results showed that the ML exhibited a continuous distribution with considerable genetic variation, indicating that this diverse population is an ideal material for GWAS studies (**Figure 1**). At a seeding depth of 2 cm, the ML ranged from 0 to 16.21 mm, with an average of 4.77 mm. At a seeding depth of 5 cm, the ML ranged from 0 to 33.90 mm, with an average of 5.61 mm. At a seeding depth of 7 cm, the ML ranged from 0 to 46.00 mm, with an average of 8.36 mm. In addition, we observed the ER. The results showed that under at 5 cm and 7 cm soil coverings, there were differences in the ERs among the different varieties. At a seeding depth of 5 cm, the ER ranged from 20% to 100%, with an average of 82.36%. At a seeding depth of 7 cm, the ER ranged from 5% to 100%, with an average of 68.88%.

Correlation analyses showed that ML exhibited a significant positive correlation among the three sowing depths: the correlation coefficients were 0.60 between 2 cm and 5 cm soil depth, 0.67 between 5 cm and 7 cm soil depth, and 0.71 between 2 cm and 7 cm soil depth. Additionally, the ERs at 5 cm and 7 cm sowing depths also exhibited a positive correlation, with a correlation coefficient of 0.46 (**Figure 1A**). In addition, independent samples t-test results indicated that there were significant differences in ML and ER under different sowing depths. A comparison of ML and ER under different sowing depths revealed that the ML followed the order of 7 cm > 5 cm > 2 cm, while the ER was higher at 5 cm than at 7 cm (P < 0.05) (**Figure 1B**). This indicates that as the sowing depth increases, the ML also increases, but the seedling ER decreases.

### 3.2 Genome-wide association analysis and candidate gene identification

The sequencing data of the materials were input and filtered, yielding 711,268 high-quality SNP markers distributed across 12 chromosomes (**Supplementary Figure S1**). The PCA results indicated that the first 20 principal components accounted for a substantial proportion of the genetic variance, exhibiting a decreasing trend, whereas the 10th and subsequent components explained less than 2% of the genetic variance (**Supplementary Figure S2**). Therefore, in this study, the group structure was corrected by principal component analysis, using the first 10 principal components as covariates. Calculations were performed using the MLM.

**Figure 2:**
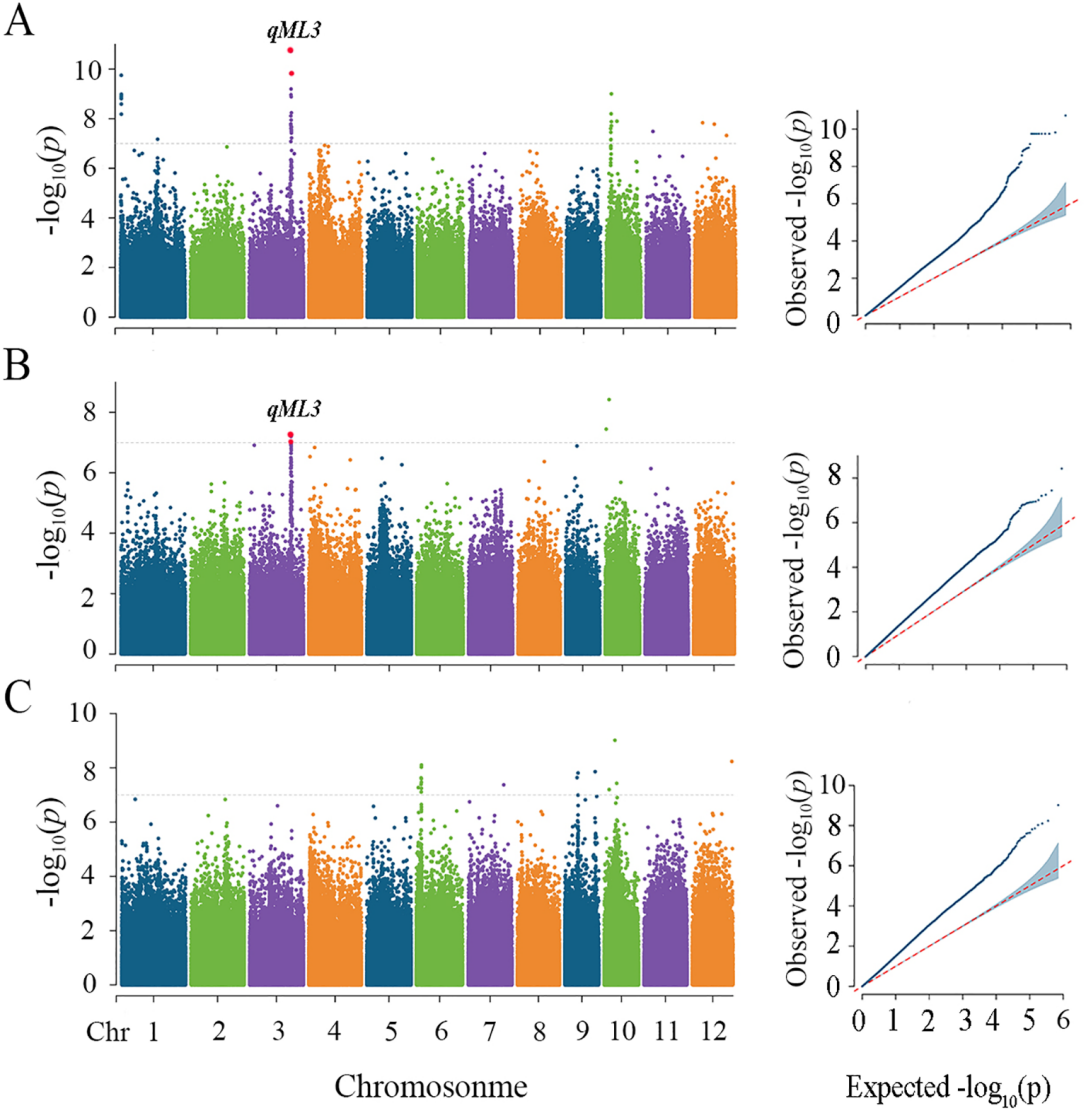
Genome-wide association studies for mesocotyl length among three deep sowing depths. Manhattan plots and Quantile-quantile plots of mesocotyl lengths in soil culture with a sowing depth of 2 cm **(A)**, 5cm **(B)** and 7cm **(C)**. Negative log10-transformed p values from a genome-wide scan are plotted against position on each of 12 chromosomes. The gray horizontal dashed lines indicate the genome-wide significance threshold. The red dots represent SNPs that were detected at both depths.

Genome-wide linkage disequilibrium (LD) attenuation in cultivated rice has been reported to occur within a range of 100–200 kb (Huang et al., 2010). We defined the region 200 kb upstream and downstream of the significant SNP as the QTL region associated. A total of six unique loci were detected, which explained the phenotypic variations ranged from 6.96% to 8.48% (**Table 1**). For ML, two significant loci were detected on chromosomes 3 and 10 at a sowing depth of 2 cm, one significant locus was detected on chromosome 3 at a sowing depth of 5 cm, and 1 significant locus were detected on chromosome 6 at a sowing depth of 7 cm, collectively explaining 6.96% to 7.54% of the phenotypic variation (**Figure 2**, **Table 1**). For ER, one significant locus was detected on chromosomes 5 at a sowing depth of 5 cm, one significant locus was detected on chromosome 4 at a sowing depth of 7 cm, with explanation rates of 8.4% and 8.48%, respectively (**Supplementary Figure S3**; **Table 1**). Among them, *qML3* detected under the 2 cm sowing depth and *qML3* detected under the 5 cm sowing depth are located at the same position in the genome, representing co-localized loci. For ease of description, we will refer to them collectively as *qML3* in the following text.

**Table 1:** Candidate QTLs identified from genome-wide association analysis.

Treatment	QTL	Chr	Position (Mb)	*P* value	R² (%)	Co-location QTL	Reference
Stress (2cm)	*qML3*	3	28.03-28.43	1.5059e-10	7.54	*qml3, qML3, qML3*	Cao et al., 2002; Niu et al., 2019; Huang et al., 2022
Stress (2cm)	*qML10*	10	3.29-3.69	9.9445e-10	6.96		
Stress (5cm)	*qML3*	3	28.03-28.43	5.6478e-08	7.19	*qml3, qML3, qML3*	Cao et al., 2002; Niu et al., 2019; Huang et al., 2022
Stress (7cm)	*qML6*	6	3.14-3.54	7.9207e-09	7.09		
Stress (5cm)	*qER5*	5	2.61-3.01	3.3156e-08	8.4		
Stress (7cm)	*qER4*	4	0-0.21	1.5602e-08	8.48		

ML, Mesocotyl length; ER, Emergence Rate; QTLs, quantitative trait loci; Chr, chromosome; R^2^, phenotypic variation rate; Position, 200 kb upstream and downstream of the significant SNP locus used to define the QTL interval; Co-location QTLs, QTLs for shoot length identified by the previous studies.

### 3.3 Haplotype analysis of candidate genes

**Figure 3:**
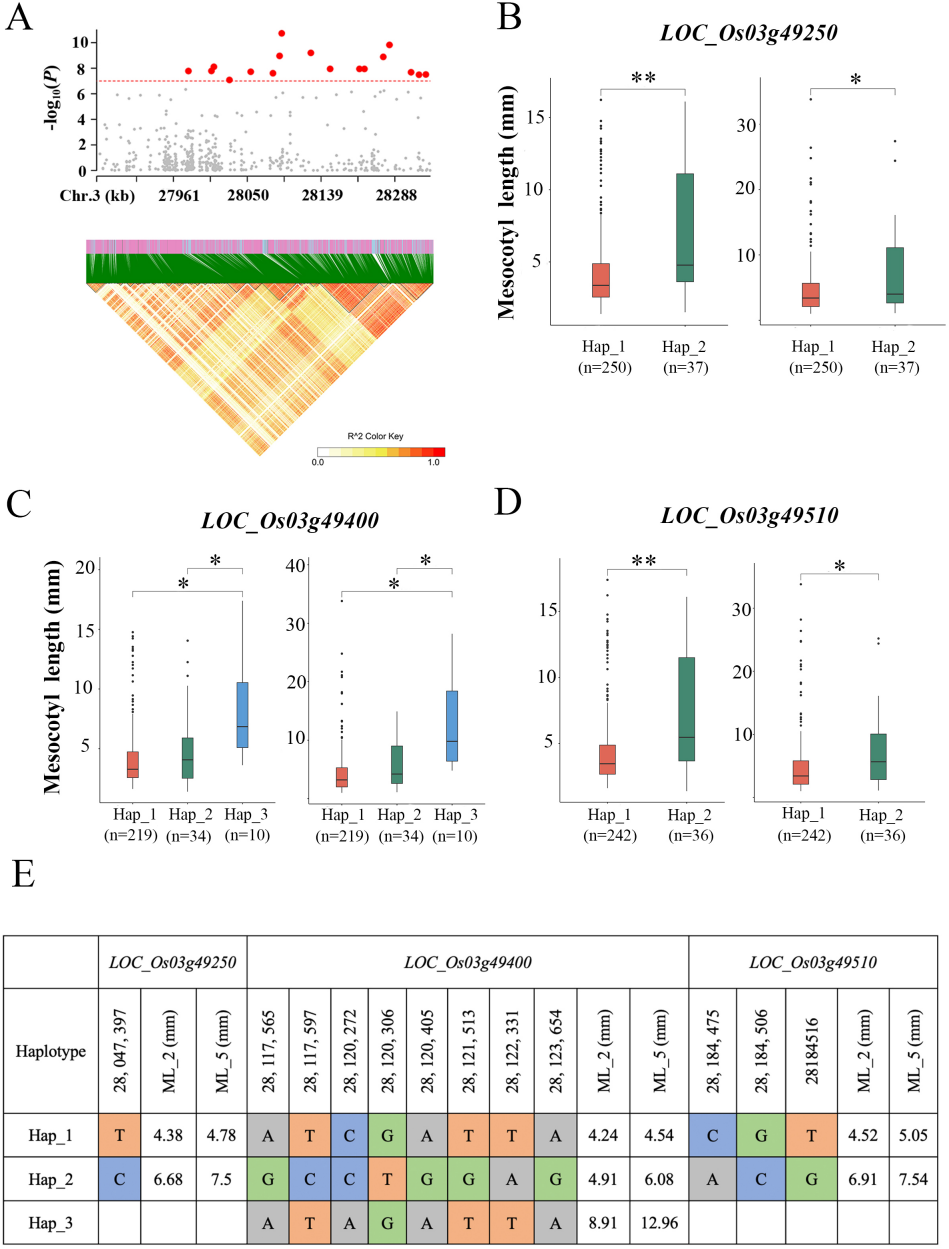
Gene structure and haplotype analysis of the candidate genes underlying *qML3*. **(A)** Local Manhattan plot (top) and linkage disequilibrium heatmap (bottom). **(B–D)** The boxplot illustrates the distribution of mesocotyl length across haplotype groups of *LOC_Os03g49250, LOC_Os03g49400* and *LOC_Os03g49510* under different sowing depths (2 cm, right; 5 cm, left). The middle line in each boxplot represents the median. P < 0.05. Significance levels are indicated by asterisks: *P < 0.05, **P < 0.01 (two-tailed t-test). **(E)** Haplotype analysis of the three genes in the region. ML_2 and ML_5: mesocotyl length under deep sowing depths of 2cm and 5cm.

By analysing the functional variants within 200 kb upstream and downstream of the peak SNPs and their LD block characteristics (**Figure 3A**), we performed a preliminary screening of candidate genes for the qML3 locus. The genetic information of 300 rice varieties has been annotated with reference to the 7th version of the MSU Rice Genome Annotation Project (Rice 7) (http://rice.plantbiology.msu.edu/) (Kawahara et al., 2013) on the rice IRGSP-1.0 genome. There were 68 genes in the *qML3* candidate QTL interval, with 44 genes remaining after the removal of putative proteins, reduced transposons, and transposon-encoding genes (**Supplementary Table S3**). Many studies have reported the association between the activities of phytohormones such as abscisic acid (ABA), BR, SL, CK, ETH, JA, GA and IAA with ML. Therefore, we focused on genes encoding phytohormone-related genes within the specified intervals, resulting in the identification of 9 candidate genes (**Table 2**).

**Table 2:** Candidate genes for gene annotation.

Gene	Functional annotation
*LOC_Os03g49050*	possible lysine decarboxylase domain containing protein
*LOC_Os03g49132*	ZOS3-16 - C2H2 zinc finger protein
*LOC_Os03g49170*	zinc finger family protein
*LOC_Os03g49250*	OsFBO16 - F-box and other domain containing protein
*LOC_Os03g49400*	ethylene-insensitive protein
*LOC_Os03g49480*	elongation of fatty acids protein 2
*LOC_Os03g49500*	ethylene receptor
*LOC_Os03g49510*	phosphatidylinositol-4-phosphate 5-kinase
*LOC_Os03g49620*	BRASSINOSTEROID INSENSITIVE 1-associated receptor kinase 1

High-confidence candidate genes for mesocotyl lengths in *qML3.*

To identify potential candidate genes, we performed haplotype analysis of nine genes based on non-synonymous mutations in the coding region of each gene. Among these genes, 2 genes showed no significant phenotypic differences between their different haplotypes (**Supplementary Figure S4**), and 4 genes had only one haplotype. Of particular interest, 3 genes (*LOC_Os03g49250, LOC_Os03g49400* and *LOC_Os03g49510*) showed significant phenotypic differences (P < 0.05) between their different haplotypes (**Figure 3**). For *LOC_Os03g49250*, one major single-base variations in the CDS region that cause amino acid substitution. The lines carrying Hap_2*^LOC_Os03g49250^* exhibited significantly longer ML than those carrying Hap_1*^LOC_Os03g49250^* under the two sowing depth conditions (P < 0.05) (**Figures 3B, E**). For *LOC_Os03g49400*, there were eight major single-base variations in the CDS region that cause amino acid substitution. The lines carrying Hap_3*^LOC_Os03g49400^* exhibited significantly longer ML than those carrying Hap_1*^LOC_Os03g49400^* and Hap_2 *^LOC_Os03g49400^* under the two sowing depth conditions (P < 0.05) (**Figures 3C, E**). For *LOC_Os03g49510*, there were three major single-base variation in its CDS region that caused amino acid substitution, and ML exhibited significantly different between the two haplotypes identified based on this variation. Under the two sowing depth conditions, the lines carrying Hap_2 *^LOC_Os03g49510^* exhibited significantly longer ML than those carrying Hap_1 *^LOC_Os03g49510^* (P < 0.05) (**Figures 3D, E**).

## 4 Discussion

### 4.1 Sowing depth affects mesocotyl length and seedling emergence rate

Soil covering depth significantly influences the elongation of rice mesocotyl and its ER. Studies have shown that as sowing depth increases, ML exhibits a significant growth trend. This adaptive elongation mechanism contributes to improving rice ERs (Zhao et al., 2018; Lu et al., 2016). Research by Lee et al. (1999) demonstrated that at sowing depths of 5, 7, and 10 cm, ML significantly increased with depth, a finding consistent with the trend observed in our experiment at sowing depths of 2, 5, and 7 cm. In addition, this pattern was also confirmed by Liu et al. (2019) who observed a significant increase in ML with increasing sowing depth at three mulch depths of 2 cm, 4 cm and 6 cm. In direct-seeded rice, deeper soil covering promotes mesocotyl elongation to facilitate seedling emergence. However, significant differences in mesocotyl elongation ability exist among different varieties: varieties with strong elongation ability can maintain a high ER under deep soil covering, while those with weak elongation ability may struggle to emerge, ultimately affecting yield. In this study, statistical analysis of seedling ERs under different sowing depths revealed that the ER at a 7 cm sowing depth was significantly lower than that at a 5 cm depth. These results indicate that sowing depth is a critical factor influencing ML and ER. Therefore, selecting an appropriate sowing depth is crucial for the practical production of direct-seeded rice.

### 4.2 *qML3* exhibits significant potential value in rice direct seeding breeding

Previous studies have identified QTLs and candidate genes associated with mesocotyl elongation in rice using linkage mapping or GWAS techniques. In this study, through GWAS analysis, we identified four QTLs related to ML and two QTLs associated with ER. Among these, a significant co-localized locus for ML, named *qML3*, was detected on chromosome 3 under both 2 cm and 5 cm sowing depths. This locus overlaps with previously identified ML QTLs (Cao et al., 2002; Niu et al., 2019; Huang et al., 2022), underscoring the authenticity and reliability of *qML3*. Furthermore, *qML3* may be closely related to the adaptability of mesocotyl elongation in shallow soil conditions, providing important insights into the genetic regulatory mechanisms of ML in such environments. Regarding ER, we did not detect any significant loci co-localized with ML, suggesting that the genetic regulation of ER may be more complex or significantly influenced by other environmental factors.

### 4.3 Candidate mesocotyl length genes

Using GWAS, we initially identified 68 candidate genes within the *qML3* interval that might be related to mesocotyl elongation. From these, nine candidate genes with homology to known functional genes were screened by functional annotation analysis and combined with previous studies. Further haplotype analysis showed that the haplotype variants of three genes, *LOC_Os03g49250*, *LOC_Os03g49400* and *LOC_Os03g49510*, were significantly associated with mesocotyl elongation, suggesting that they may be potential candidate genes for *qML3*.

All three candidate genes screened in this study regulate mesocotyl and internode elongation through the phytohormone signalling pathway. *LOC_Os03g49250* encodes an F-box protein, a family of proteins that play important roles in plant signalling (Yan et al., 2017). Its homologous gene, D3, was shown to specifically recognise the OsGSK2-phosphorylated CYCLIN U2 protein and promote its degradation through the ubiquitin-proteasome pathway, thereby negatively regulating rice mesocotyl elongation (Sun et al., 2018). The ethylene-insensitive protein (EIN) encoded by *LOC_Os03g49400* is a core component of the ethylene signalling pathway, and it has been shown that ethylene affects mesocotyl development by regulating the expression of JA and BR related genes via EIN2/EIL2 (Xiong et al., 2017; Jun et al., 2004). *LOC_Os03g49510* encodes phosphatidylinositol 4-phosphate 5-kinase, a family of enzymes involved in growth hormone signalling and plant developmental processes through the regulation of phospholipid metabolism (Liang et al., 2018). It was found that OsPIP5K1 and DWT1/DWL2 synergistically regulate rice growth and development through phosphatidylinositol signalling, and its functional deficiency leads to disturbances in phospholipid metabolism, triggering typical phenotypes such as plant dwarfing and internode shortening (Fang et al., 2020).

During plant growth, the gradual increase in mesocotyl length is typically regulated by phytohormones through complex signaling pathways. Generally, phytohormones control mesocotyl elongation by modulating either cell division or cell expansion (Zhan et al., 2019). In this study, the three identified candidate genes were found to be associated with the biosynthesis and signalling pathways of plant hormones, suggesting their potential involvement in mesocotyl elongation by regulating the expression of hormone-related proteins. The molecular pathways by which the proteins encoded by these genes regulate mesocotyl elongation growth and the correlation (positive or negative) between changes in their expression levels (up- or down-regulation) and the rate of mesocotyl elongation need to be further investigated. In the following work, we will use molecular biology techniques such as gene editing, genetic transformation and DNA insertion to systematically verify the biological functions of these candidate genes in mesocotyl elongation.

## 5 Conclusions

This study utilized GWAS to analyze the ML and seedling ER of 300 rice germplasm resources. By analyzing two traits, ML and ER, a total of six QTLs were identified. Among them, the ML-related locus *qML3* was detected under two different sowing depths and contributed the most to phenotypic variation, indicating its stable genetic effects across multiple environmental conditions. Further prediction of candidate genes for *qML3* identified three potential candidate genes associated with ML: *LOC_Os03g49250*, *LOC_Os03g49400*, and *LOC_Os03g49510*. The functions of these genes require further validation, but their potential mechanisms provide new research directions for the regulation of mesocotyl elongation. In the future, these genes are expected to be applied in molecular breeding for ideal rice plant types, offering theoretical foundations and genetic resources for the improvement of direct-seeded rice.

## Data Availability

The datasets presented in this study can be found in online repositories. The names of the repository/repositories and accession number(s) can be found in the article/**Supplementary Material**.
