# Supplementary material for: Identification of key genes associated with mesocotyl length through a genome-wide association study in rice
Source: Front Plant Sci. 2025 May 13;16:1546580. doi: 10.3389/fpls.2025.1546580 (PMC12106522; doi:10.3389/fpls.2025.1546580)
Supplement: Supplementary file 1 [file Table1.docx]

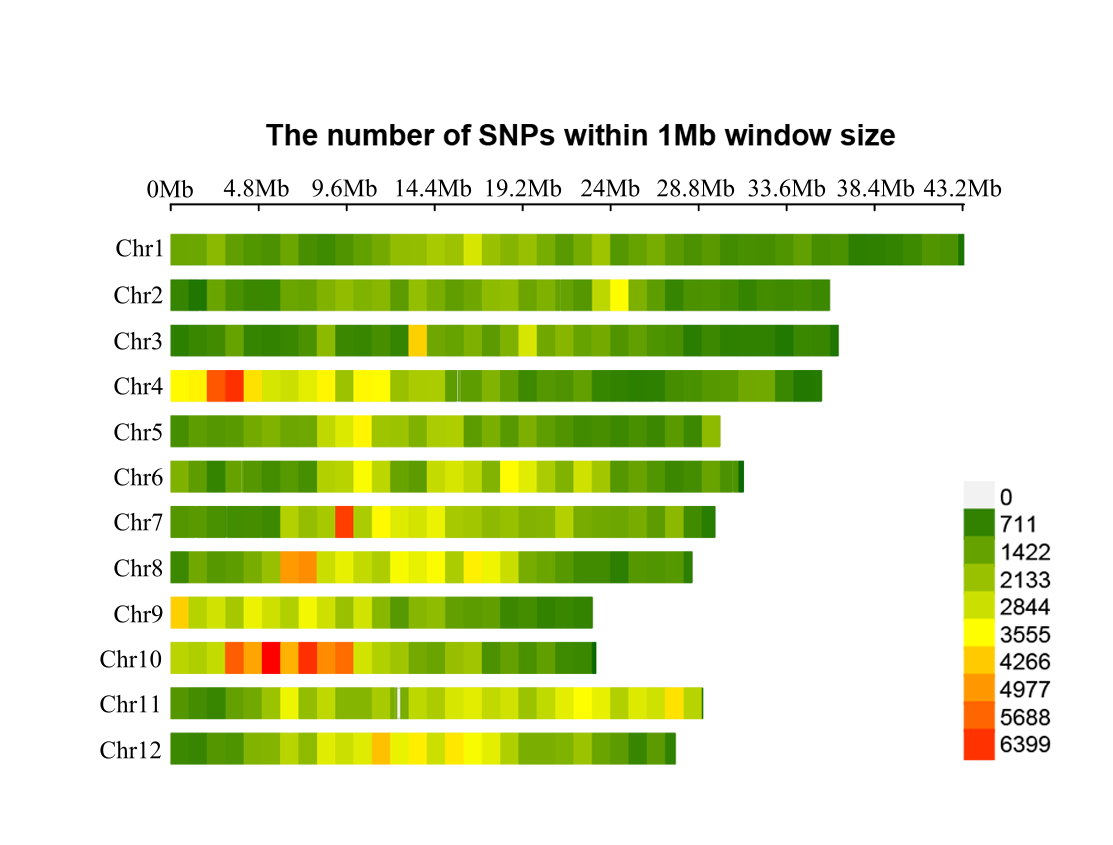
**SUPPLEMENTARY FIGURES**


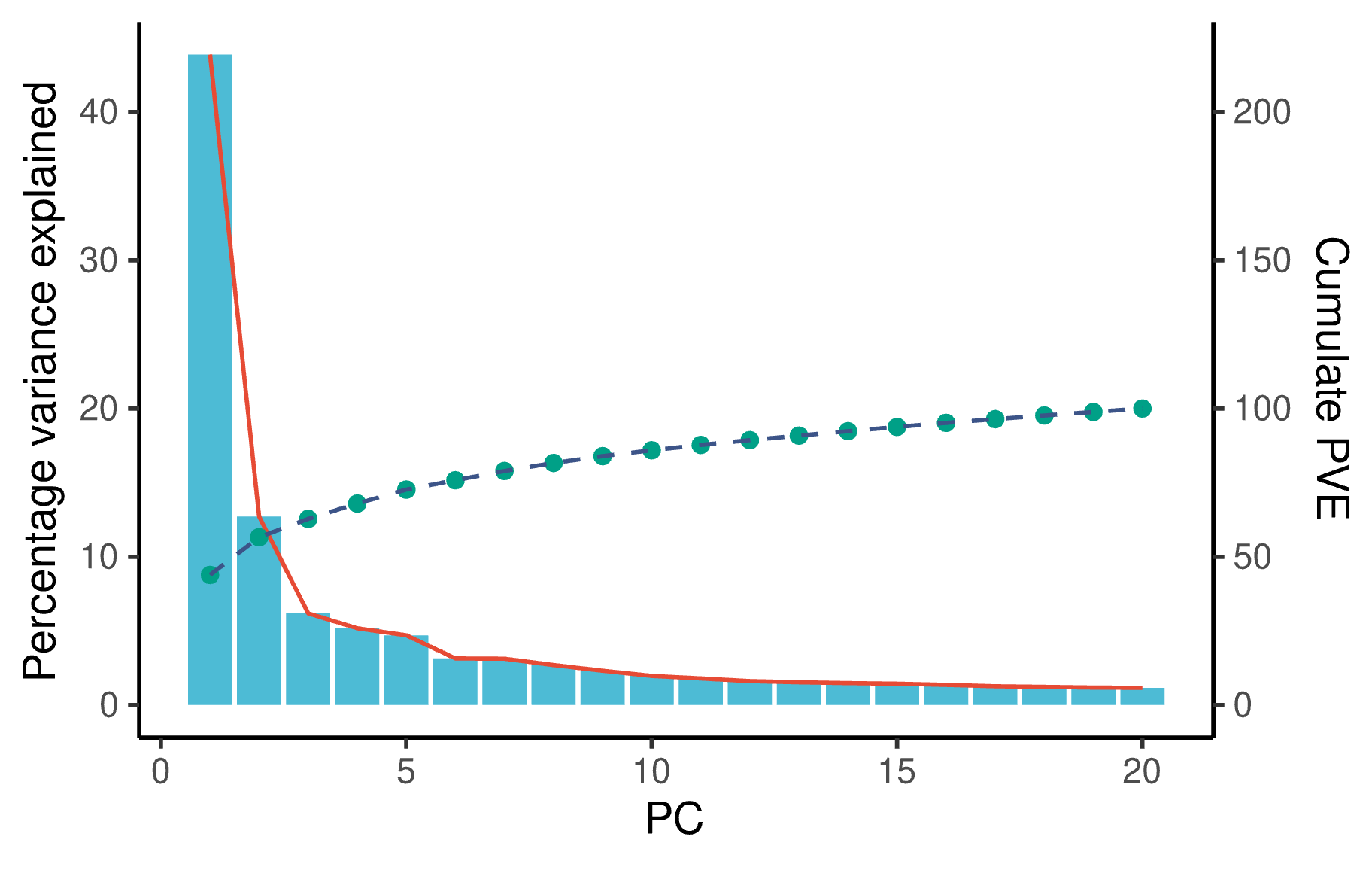
**Figure S1.** Density heatmap of SNPs on 12 chromosomes.

**Figure S2.** The percentage of genetic variation explained by each of the first 20 principal components (PCs)

**
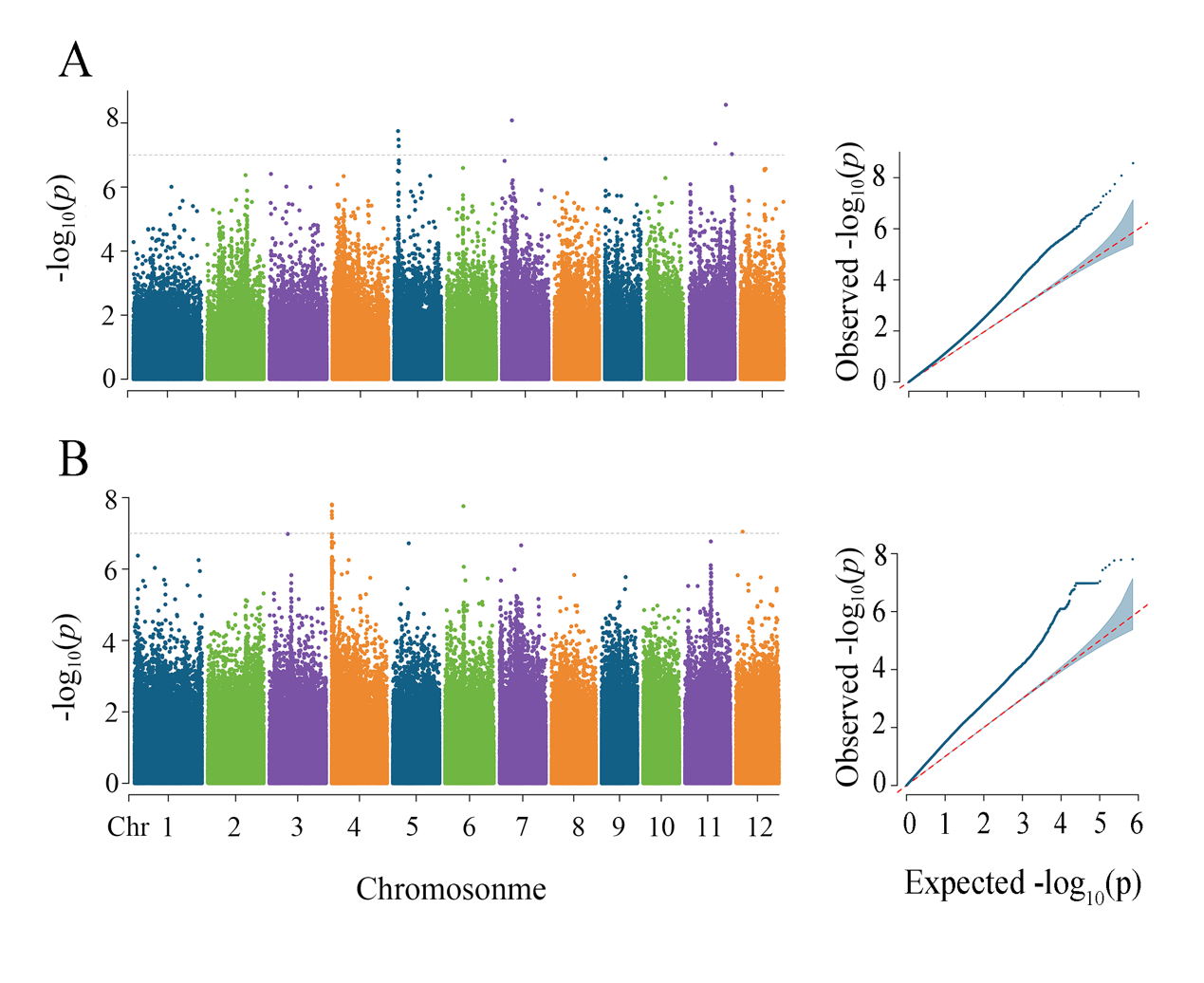
Figure S3.** Genome-wide association studies for Emergence Rate among two deep sowing depths. Manhattan plots and Quantile-quantile plots of Emergence Rate in soil culture with a sowing depth of 5 cm (A) and 7cm (B). Negative log10-transformed p values from a genome-wide scan are plotted against position on each of 12 chromosomes. The gray horizontal dashed lines indicate the genome-wide significance threshold.

**
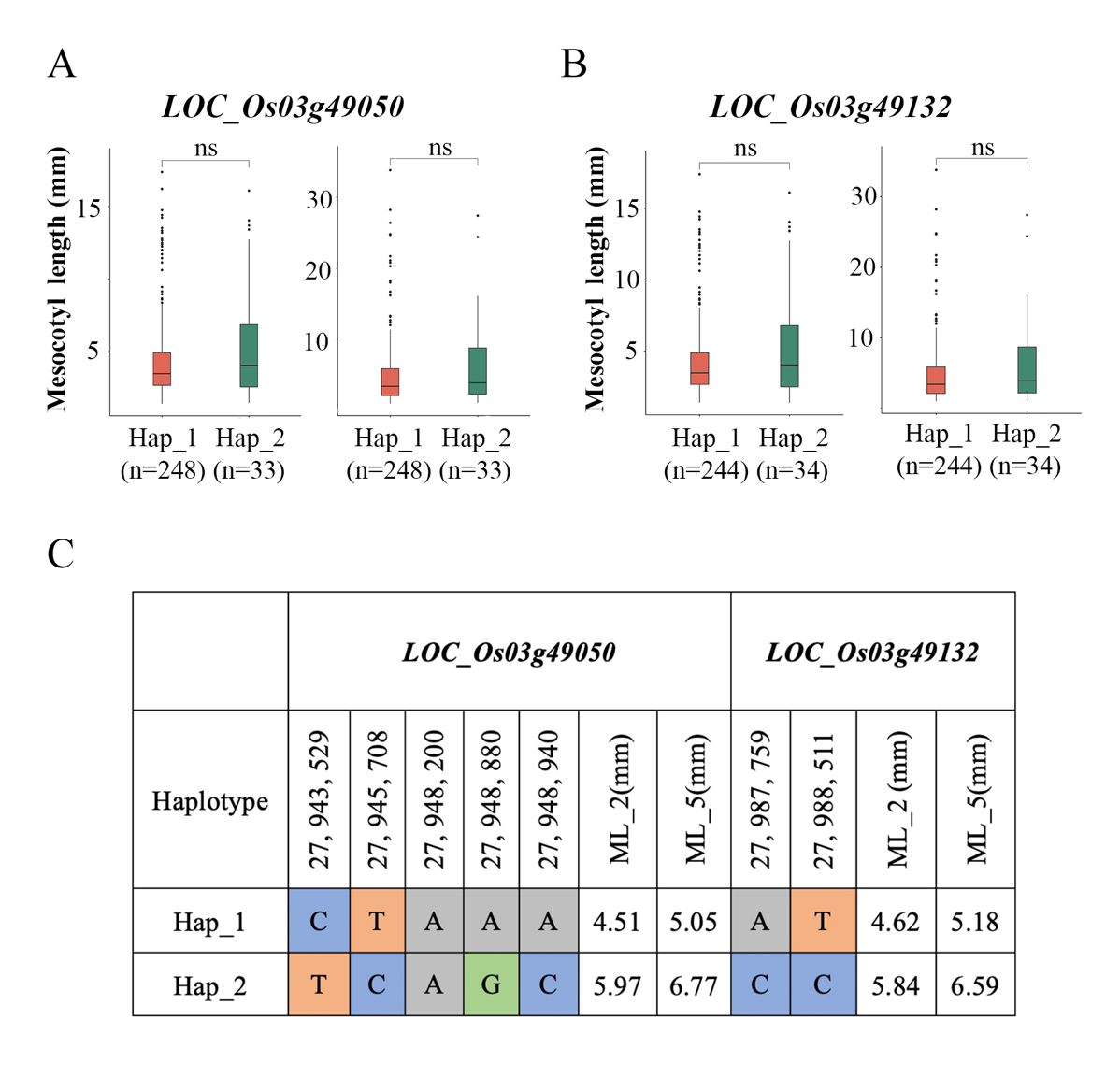
Figure S4.** Gene structure and haplotype analysis of the candidate genes underlying *qML3*. (A) and (B)The boxplot illustrates the distribution of mesocotyl length across haplotype groups of *LOC_Os03g49050* and *LOC_Os03g49132* under different sowing depths (2 cm, right; 5 cm, left). The middle line in each boxplot represents the median. P < 0.05. (C) Haplotype analysis of the two genes in the region. ML_2 and ML_5: mesocotyl length under deep sowing depths of 2cm and 5cm.
